# Perceptions of digitally supported home exercise for people with Parkinson's disease: A qualitative study

**DOI:** 10.1177/02692155241298859

**Published:** 2024-12-05

**Authors:** Jenny Sedhed, Hanna Johansson, Elisabet Åkesson, Erika Franzén, Breiffni Leavy

**Affiliations:** 1 Department of Neurobiology, Care sciences and Society, 27106 Division of Physiotherapy, Karolinska Institutet, Stockholm, Sweden; 2 Stockholm Sjukhem Foundation, 27103 R&D unit, Stockholm, Sweden; 3 Theme Women's Health and Allied Health Professionals, Medical unit Occupational Therapy & Physiotherapy, 27103 Karolinska University Hospital, Stockholm, Sweden; 4 Department of Neurobiology, Care Sciences and Society, 27103 Division of Neurogeriatrics, Karolinska Institutet, Stockholm, Sweden

**Keywords:** Home exercise, Parkinson disease, eHealth, qualitative

## Abstract

**Background:**

Digitally supported home exercise offers the potential to expand accessibility to rehabilitation. However, little is known about how people with Parkinson's disease experience performing home exercise programs using digital delivery.

**Objective:**

To explore and describe how people with Parkinson's disease perceive digital home-based exercise that is not supported in real-time, and how it affected their everyday lives.

**Methods:**

This study was qualitative in nature, using qualitative content analysis with an inductive approach. In-depth, individual, semi-structured interviews were held with 14 participants with Parkinson's disease.

**Results:**

Two overarching themes were formed: “Active agency in the face of uncertainty” and “The home – safe space or disability on display”. The overarching themes were formed by four themes: 1) resisting the disease - a hope and a burden, 2) interpreting mixed messages, 3) home exercise – consideration and responsibility, and 4) the social context - judgement and support. Participants with Parkinson's disease believe that home exercise enables them to actively counter the disease, and this belief serves as both a source of hope but also a burden. Although perceived as advantageous, digitally supported home exercise may also incur becoming vulnerable to exposing one's disability.

**Conclusions:**

People with Parkinson's disease struggle to reconcile their beliefs about exercise with that which is encouraged or discouraged by those around them. Exercise in the home involves a dynamic interplay between achieving self-directed goals while trying to balance social consideration and maintaining integrity of identity.

## Introduction

Exercise plays an important role in managing disease symptoms in Parkinson's disease. Strong evidence supports both short^1^ and long-term effects^2^ of physical activity and exercise, which emphasizes the importance of maintaining a physically active lifestyle throughout the disease course. Nonetheless, people with Parkinson's disease tend to be less active compared to healthy peers.^3–5^

Parkinson's disease is the fastest growing neurological disorder to date.^6^ Healthcare systems, as currently organized, are generally not resourced to provide continual rehabilitation for the majority of patients in need. Alternative care models need therefore to be developed, such as those involving digital options, to increase accessibility to rehabilitation for this growing Parkinson's disease population. People with Parkinson's disease, their family members, and healthcare professionals increasingly view digital interventions as an opportunity to enhance the provision of rehabilitation.^7^ There is growing meta-analytic evidence that eHealth has the potential to improve motor impairments in Parkinson's disease.^8^

The need for eHealth solutions accelerated during the Covid-19 pandemic. Programs initially intended to be clinic-based were adapted to digital home-based formats.^9–11^ Of the qualitative studies which have sought to explore participant experiences of digital home-based interventions, several involve synchronous training where the instructor supported participants during each session.^9–11^ Fewer studies have explored the experiences of asynchronous exercise, where participants are not supported in real-time,^12,13^ even though this alternative is plausibly a less time-consuming and more cost-effective model of healthcare delivery. A comparison between the two models reports that, when not supported in real time, home exercise is performed with lower intensity and adherence.^12^ In order to improve the design and acceptability of digital home-based exercise programs, we first need to increase our understanding of how people with Parkinson's disease experience performing a digital home-based intervention. The aim of this study was to explore the perspectives of people with Parkinson's disease who had participated in a digital home-based exercise program, which was not supported in real-time, and how it affected their everyday lives.

## Methods

### Design and participants

This study aimed to explore and describe how people with Parkinson's disease experienced conducting exercise at home, supported by eHealth methods. A qualitative methodological approach using individual interviews was applied, as this method is appropriate when aiming to capture and describe the lived experience.^14^ Fourteen participants who successfully completed a prior feasibility study,^15^ during which they engaged in ten weeks of motor-cognitive home exercise using a tablet and an app, were asked to be interviewed. The home exercise program was tailored to each participant's baseline motor and cognitive capacity. Exercises were progressed during the 10-week intervention period, primarily at the 3-week and 5-week timepoint. All participants received support from the research team through the digital platform, using messaging and video chat, whenever questions or comments regarding their exercise program arose. Additionally, participants received messages from the research team throughout the intervention period and had a video call in week five, to discuss their progress. Participants were screened for eligibility prior to inclusion in the previous study. They were eligible for inclusion if they (1) had a diagnosis of Idiopathic Parkinson's disease from a neurologist, (2) were ≥50 years, (3) were assessed as Hoehn and Yahr Stages 1–3, (4) were able to ambulate indoors without a mobility aid, and (5) had a stable anti-Parkinson's medication regime three months prior to inclusion. People were excluded if they had cognitive difficulties, as defined by a score ≤21 on Montreal Cognitive Assessment (MoCA^©^), did not have access to Wi-Fi, had significant or uncorrected impairment of hearing or vision. Included participants (Table 1) were equally distributed between genders, had a mean age of 68.4 years and were Hoehn and Yahr stage 2 or 3. The majority of the sample reported having experience of technology use prior to inclusion in the feasibility study (n = 9).

**Table 1.** Participant characteristics.

ID	Sex	Age range (years)	Disease duration (years)	Hoehn and Yahr (1–5)	Experience of technology use
1	Male	50–59	2.5	2	Yes
2	Male	60–69	4	2	Yes
3	Female	60–69	6	3	Yes
4	Male	70–79	3	2	Yes
5	Male	60–69	22	3	Yes
6	Male	70–79	16	3	No
7	Female	50–59	5	2	Yes
8	Female	70–79	7	3	No
9	Male	70–79	6	3	Yes
10	Female	60–69	5	3	Some
11	Female	70–79	4	3	Some
12	Male	70–79	3	2	Yes
13	Female	70–79	9	3	Yes
14	Female	70–79	4	2	Some

### Data collection

All 14 participants who completed the feasibility study consented to the interview. The interviews were conducted during the spring of 2022 by the first author JS, who had provided online support to participants throughout the feasibility study. Open-ended questions were asked in accordance with a semi-structured interview guide (see Multimedia Appendix 1). The interview guide was constructed around six predefined themes: living with Parkinson's disease, living a physically active life, the experience of exercising using technology, the experience of exercising at home using a digital tool, experiences, and insights regarding exercise at home, and physical activity in daily life. The interviews were conducted in participants’ homes, at Karolinska Institutet facilities or a health care provider's facility, according to each participant's preference. Interviews were held in Swedish and recorded using a Dictaphone (Olympus VN-741PC) and ranged between 55–110 min (average time was 81 min). Field notes were written after each interview. The interviews were transcribed verbatim by first author JS, with the help of two research assistants. Following the first interview, authors JS and BL discussed and evaluated the interview technique and the interview guide. Minor changes to the sequence and formulation of certain questions were made. Further details in relation to data collection are reported in the consolidated criteria for reporting qualitative research (COREQ) checklist^16^ (see Multimedia Appendix 2).

### Data analysis

Qualitative content analysis was used with an inductive approach, as this method is viewed to be valid and systematically captures key themes from text data.^17^ Each transcript was read repeatedly by JS, who thereafter identified meaning units. In order to validate the process, authors BL and HJ independently read and identified meaning units from two transcripts and compared to those identified by JS. Further, coding and categorization was conducted by JS on multiple occasions, revisiting the process continuously. Following the first coding, the process was discussed and reviewed with authors BL and HJ. Initial categories were formed by authors JS, HJ and BL and later discussed together with all authors. The analytical process from sub-themes, main themes to the final the overarching themes was discussed during a series of peer debriefing meetings with all authors to ensure unity and conformability of the analysis.^17^

## Results

**Figure 1.**
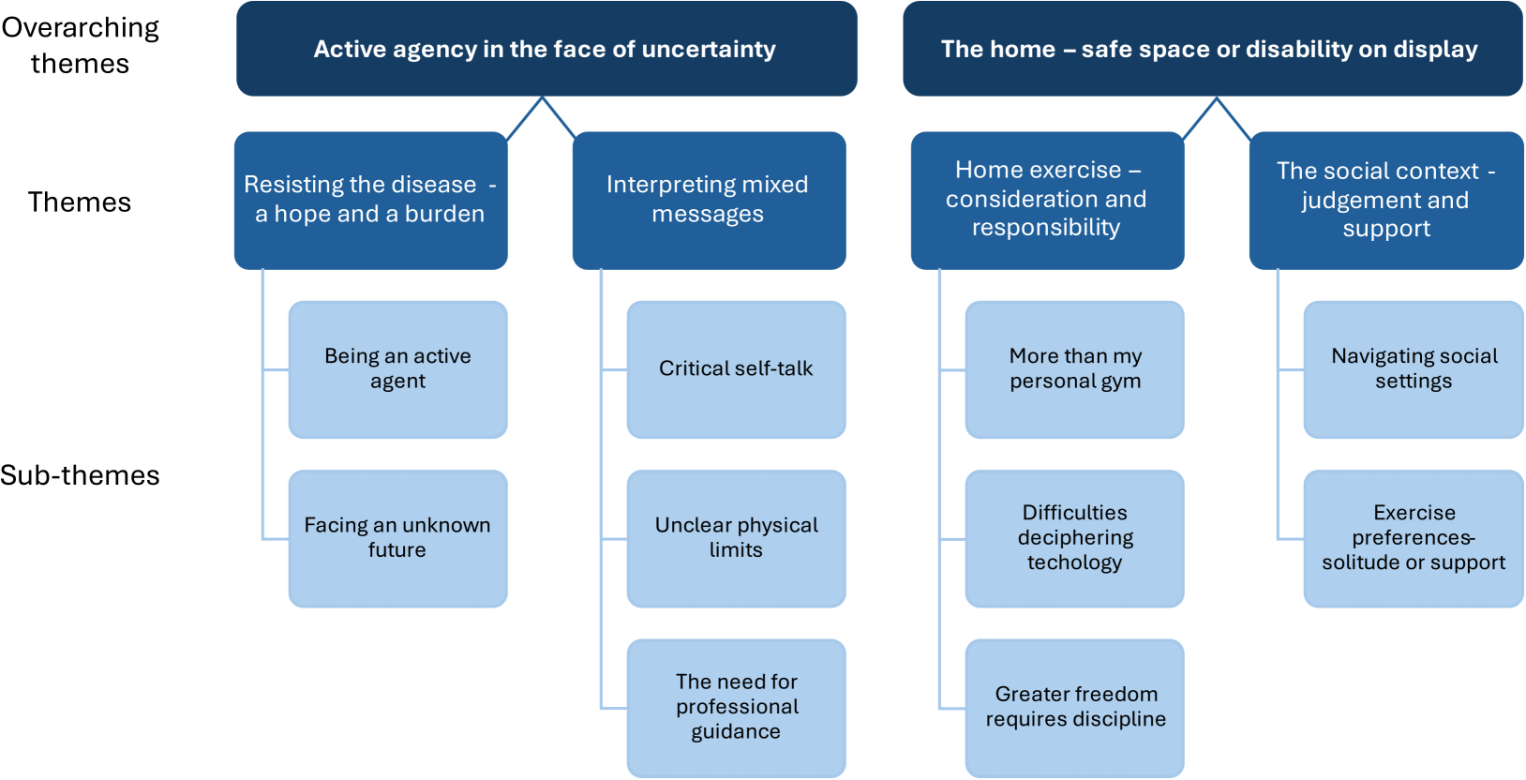
An overview of overarching themes, themes, and sub-themes.

The qualitative content analysis resulted in the formation of ten sub-themes, four themes and two overarching themes: “Active agency in the face of uncertainty” and “The home – safe space or disability on display”, see Figure 1. *Active agency in the face of uncertainty* concerned how home exercise was viewed as a means by which to actively engage in countering the disease – a source of both hope and burden. People with Parkinson's disease are forced to reconcile mixed messages concerning their physical symptoms, with the protective concerns of loved ones, and from healthcare providers, on how best to challenge their physical limits. *The home – safe space or disability on display*, captured the dynamic interplay between achieving self-directed exercise goals while trying to balance being socially considerate and maintaining integrity of identity in the home environment.

### Resisting the disease - a hope and a burden

This theme is composed of two subthemes which describe participants’ perceptions of home exercise as a means to actively resist the disease, alongside feelings of uncertainty for what the future holds.

The subtheme *Being and active agent* incorporates participants’ desire to use exercise to impede disease progression and the resultant conflicting emotions involved in resisting the disease. Participants expressed an awareness of how exercise could be used to alleviate disease symptoms, and that this was perceived as motivating. Exercise was often portrayed as being equally important as pharmaceutical treatment. However, they also described how this awareness could lead to feelings of personal responsibility and a sense of obligation to do everything in their power to slow down the course of the disease. These perceptions gave rise to conflicting emotions concerning feeling hopeful and driven to influence the disease, while also experiencing the inherent burden of being personally accountable for their disease trajectory. The experience of being an active agent in fighting the disease was therefore a source of hope, but also a source of burden.

“*Before, you could do that…choose whether you could be fit or in good physical shape or not…and still get away without exercising that much. I’ve been in a situation over the past three years where I have to exercise and keep moving…It's a basic requirement to slow down the (disease) development…so it's like, you could call it motivation or punishment.”*

*Participant 1*

Captured in the subtheme *Facing an unknown future* are participants’ feelings of apprehension for what the future holds alongside a resistance and reluctance to succumb to the disease. After receiving a Parkinson's disease diagnosis, participants expressed both a need to reconcile with their ‘new life’, and an unwillingness to accept how the disease affected their everyday lives. Some participants described using coping strategies such as keeping busy, and consciously shifting focus from themselves to external aspects of life to contend with their deteriorating illness.

“*You have to get your brain to accept that you need help. That do-it-yourself way of thinking has been hard to get rid of. That I won’t be able to take care of myself, I have put that thought aside, it will be what it will be.”*

*Participant 13*

### Interpreting mixed messages

The second theme highlights the conflict between recognizing the benefits of exercise while also struggling to perform it. This theme includes three subthemes: critical self-talk, unclear physical limits, and the need for professional guidance.

The subtheme *Critical self-image* incorporates findings concerning participants’ self-image and distrust of the body. Many people recognized the importance of intrinsic motivation and self-discipline when exercising at home. Despite their awareness of the benefits of exercise, and their intention to engage in it, participants found it challenging to follow through with home exercise routines. The use of harsh rhetoric was common in their self-description, whereby they referred to themselves as ‘lazy’ or ‘complacent’. Participants described how fluctuations in symptoms, lack of initiative, and fatigue undermined their everyday activities, leading to distrust towards their bodies and a dubious self-image. Notably, these undesired symptoms appeared to be interpreted as character flaws, as opposed to being grounded in the disease.

“*It's like I said, you can manage certain things like you used to, you can manage to do the vacuuming but then you don’t have the energy left to do anything else that day, more or less. It's a psychological problem really, more than something physical I think, that you lose the initiative to do things. It's like, I can’t put my finger on it really, but it's like you have no drive to do things, you just don’t feel motivated enough maybe”*

*Participant 12*

The subtheme *Unclear physical limits* deals with how the unpredictable nature of Parkinson's disease made it difficult for participants to anticipate their physical limits. The importance of finding balance between activity and rest was stressed, as well as determining an appropriate level of difficulty when exercising. Although participants had, since disease onset, experienced declines in physical capacity, their desire to be physically challenged had not diminished. This unmet need to physically exhaust the body led to frustration. Certain participants expressed how the exercise program could have been more physically demanding.

Additionally, participants expressed frustration with relatives, friends, and healthcare professionals who urged them towards caution, as opposed to challenging their physical limits. A clear conflict arose between participants’ aspirations, and the advice of others, which guided them towards a more restrictive and guarded approach. They also expressed a longing for support from family and friends as well as desire for them to receive education concerning disease complexity.

“*You feel, you feel lazy, but you just don’t have the energy to do anything. It's not about not wanting to, it's more about not being able to. And if you work too much despite your body and decide that you are sure as hell going to ski ‘Vasaloppet’ (ski race) or something like that, well then you will have to pay for that, there is a price to pay.”*

*Participant 5*

The subtheme *The need for professional guidance* concerns the perceived need for support from healthcare professionals to continually monitor the performance of home exercise. Participants emphasized the importance of having a professional who was engaged in their development ‘on the other side’ of the screen. They expressed how continuous feedback during home exercise was necessary if individual progress was to be made. Furthermore, they described feeling a lack of trust in their own bodies, which made it challenging for them to distinguish whether certain exercises were more advantageous than others. This amplified their desire for professional feedback to assure them that exercises had been performed correctly.

“*A lot of it is in your head, that someone knows what suits me, what a reasonable program is for me. Should I have to work that out? What can I do? What are my limits? What is good for me? Because I have done exercise where I made things worse, because I didn’t do it the right way”*

*Participant 14 *

### Home exercise – consideration and responsibility

The third theme emphasized the significance of the home environment and digital competence in facilitating effective exercise routines.

The subtheme *More than my personal gym* highlights complexities which arise when the home − a shared space and a safe haven, becomes a place for exercise. Important physical aspects of the home environment involved the availability of adequate space and equipment to enable exercise. The need for peace and quiet was also stressed in order to achieve focus during the exercises, which often included cognitive elements. Furthermore, aspects of co-habitation appeared to strongly influence how home exercise was perceived. Specifically, being considerate to the needs of a partner or child could interfere with participants’ ability to exercise as planned at home. When participants lived with others, exercising at home also incurred becoming vulnerable to exposing their disability. In line with this experience, some people who lived alone expressed that being unobserved while exercising felt liberating.

“*You can do the movements, and you don’t need to compare yourself to anyone, like if you shake a lot or if you are really stiff, so nobody needs to see that, because you are at home.”*

*Participant 10*

*Difficulties deciphering technology *concerned the need for technology which supported digital home exercise to be user-friendly and easily interpretable. Participants described how difficulties interpreting and handling technical platforms had become apparent, since the onset of their disease. They also expressed clear opinions regarding the exercise application, describing it as lacking in certain aspects. Exercising at home and being solely responsible for technical issues that arose strengthened participants’ expectations that technology operated as intended. Additionally, participants acknowledged the need for a certain level of digital literacy in order to navigate the application and interpret arising errors.

“*You become completely dependent on the fact that what you expect to show on the screen, actually shows on the screen. Something else can’t come up that you have to click away……and it's enough with one error, because the chain is only as strong as its weakest link*”

* Participant 4*

The subtheme *Greater freedom requires discipline* involves perceptions of digitally supported home exercise as advantageous, while also incurring the need for self-motivation. The flexibility of being able to choose when to exercise, throughout the day and week, was viewed as advantageous and conducive to exercise adherence. The freedom to postpone an exercise session during times of symptom exacerbation was also seen to enhance exercise adherence. Nevertheless, participants acknowledged that with this freedom came the need for greater discipline and motivation, which they feared could be a pitfall when performing unsupervised exercise.

“*I don’t know, with home exercise, I end up thinking that…If you are self-motivated there is no problem with home exercise. You need to set goals that you stick to. If you don’t do that, but just go on how you feel, that's dangerous…Then home exercise doesn’t work”*

*Participant 1*

### The Social context - judgement and support

This theme describes the social space and environment which was experienced as either supportive or isolating.

The subtheme *Navigating social settings* deals with perceptions of loneliness and difficulties navigating social contexts. Participants expressed feelings of anxiety and even strong aversion to social gatherings, where specific Parkinson's disease symptoms such as tremor while eating, vocal weakness and stiff posture were described as being a ‘social handicap’. Certain participants experienced a judgmental gaze from onlookers and were self-conscious of being perceived as being under the influence of drugs or alcohol. The resultant avoidance of social contexts sometime led to feelings of loneliness and isolation. For certain participants, avoiding social situations came with a sense of relief.

“*I don’t even go out to eat because, when I eat, my hands they shake then, when I’m about to eat, so sometimes it feels like people are watching, I don’t know, sometimes that feels that really tough”*

*Participant 3*

The subtheme *Exercise preferences- solitude or support,* captured diverse experiences and preferences regarding exercising alone or with others. The merits and drawbacks of digitally supported exercise alone at home were weighed against participants’ previous experience of a group exercise context. Certain participants preferred to exercise alone, as they saw exercise in a Parkinson's group context as a painful reminder of the looming threat of future disability. Others simply had no interest in sharing this activity with others.

“*I have, in a way, avoided groups. It's not only to do with Parkinson's, but to a certain extent, because I feel like I don't do the movements as quickly as others and would feel a little bit stressed, I think, that… so it's not so good for me”*

*Participant 13*

Conversely, certain participants with previous experience of exercising with others with Parkinson's disease, felt supported and safe in that context, as they believed their peers shared the lived experience inherent to the disease. This belief incited feelings of solidarity − a sense of being “in the same boat”. For these participants, witnessing others battle with symptoms was perceived as motivating and inspirational.

“*I think so, that there is more connection, that we are in the same boat. It was very helpful for me anyway, like I said, that I had that opportunity.”*

Participant 14

## Discussion

This qualitative inquiry explored how people with Parkinson's disease perceived digitally supported home exercise, which was not delivered in real time, within the context of their everyday lives. The two overarching themes capture the multiple dilemmas faced by this group when exercising alone in the home environment. Whereas home exercise was viewed as enabling a sense of active agency, these beliefs also occurred alongside feelings of burden and confusion due to conflicting messages. Additionally, when the home becomes the exercise domain, social considerations need to be accounted for, and personal integrity may be threatened.

Participants in the current study demonstrated an awareness of how home exercise could help manage their symptoms. Although they acknowledged this belief as a source of hope, they also expressed how it was accompanied by burdensome feelings of responsibility. For some, being solely responsible for performing exercise at home served to accentuate these feelings of burden. For people living with a chronic and degenerative condition, such as Parkinson's disease, continual self-care is imperative. Self-care is, however, challenging and influenced by factors such as motivation, confidence, and cognitive and functional abilities.^18^ Self-regulation theory postulates that self-efficacy is central to initiating and maintaining exercise behavior, while goal-setting can serve to motivate it.^19^ Performing exercise can also be seen as a means to maintain normality, and to cope with the continual changes in everyday life with Parkinson's disease.^20^ The unpredictable nature of the disease course was another source of worry. Participants described how they tried to reconcile with their new life, while also resorting to strategies to avert thoughts of the future. Fear of disease progression is highly prevalent among people with Parkinson's disease and is associated with higher levels of anxiety and lower self-efficacy.^21^

Participants struggled to make sense of their own ideas about physical activity and exercise and reconcile them with that which family members encouraged, and that which healthcare professionals advised. Notably, participants appeared to internalize non-motor symptoms as personal character flaws rather than recognizing them as a natural consequence of the disease. Although research has increased the understanding of how non-motor symptoms negatively affect exercise engagement,^22^ these symptoms are still underrecognized and misunderstood among patients and their caregivers.^23^ It is plausible that these evoked feelings of inadequacy could negatively affect participants’ self-efficacy and exercise engagement. This finding is therefore of particular relevance for healthcare professionals who promote and support home exercise.

Participants in this study expressed feelings of distrust in their bodies, which coincided with difficulties understanding their own physical limits. Although they explicitly stated an urge to feel physically exhausted through exercise, they struggled to find a balance between activity and rest. Consequently, they stressed the importance of receiving guidance from healthcare professionals. These findings regarding experienced barriers to exercise involvement build upon those by O’Brien et al., where apathy, fatigue and belief in a finite energy quota are recounted.^22^

This study highlights dilemmas which may arise when the home – a safe haven, becomes the main domain for exercise. While generally viewed positively, self-managing the disease at home through exercise could also encroach on the personal space of family members and leave participants feeling exposed. Interpretation of these findings can be strengthened by a conceptual framework from the field of environmental gerontology – the *Person-environment exchange* - which accounts for the home as being more than just a physical space, by describing its behavioral and experiential aspects.^24^ Interpreted through the lens of person-environment exchange theory, tensions may arise when a person's *agency* (in this case the intention to exercise at home) may be hindered by emotional aspects of *belonging* (in the social context with others).^25^ Whereas a person's *agency* may decline due to progressive disease, the relative significance of *belonging* appears to increase across the life span.^26^ That these dynamic processes influence a person's sense of *autonomy* and their sense of *identity,* is important to consider when prescribing and supporting home exercise programs for people with Parkinson's disease.

Our findings provide first-hand evidence for how people with Parkinson's disease value the importance of user-friendly digital platforms, alongside access to technical support when availing of eHealth-supported home exercise. High useability is previously recognized to facilitate the implementation of technology for disease management.^27,28^ Our results also demonstrate awareness of the need for digital literacy, from the stakeholder's perspective, when implementing this mode of exercise delivery.

Perceptions of the relevance of the social context during exercise were diverse. Our findings add to existing evidence for the socially isolating consequences of Parkinson's disease symptom progression.^29–33^ and how this group often values social support^34,35^ and exercise alongside peers who share a similar diagnosis.^31,36^ For certain individuals, however, the social context requires careful navigation and unobserved exercise in the safe space of the home, provides a means of avoiding what they perceive as the judgmental gaze of others.

There are factors to consider that may have affected trustworthiness. The first author (JS) both supported the intervention during the feasibility study and later performed the interviews, which may have influenced participants’ responses and affected study findings. However, the author's in-depth insight of the intervention may have bolstered throughout the systematic analytical process. By having this preunderstanding as an interviewer, participants could more effectively articulate their experiences and challenges that occurred during the intervention period. This, in turn, could allow them to extend their reflections to broader concepts. To ensure transparency of the analysis, quotes were chosen carefully to best represent each sub-theme in the results. Member checking was not performed in the current study, which could have further enhanced the credibility and trustworthiness of the findings.

In conclusion, our findings illustrate how people with Parkinson's disease perceive that digitally supported home exercise enables them to actively counter the disease, and how this belief is both a source of hope and a burden. When performing exercise alone in the home, this group faces uncertainty while reconciling their own thoughts about exercise with that which is encouraged or discouraged by family members and healthcare professionals. Performing digitally supported exercise in the home may involve a dynamic interplay between achieving self-directed goals while trying to balance social consideration and maintain integrity of identity.

**Clinical messages**

- Performance of home exercise is perceived as a way to actively resist disease progression in Parkinson's disease – a belief which provides hope but is also a burden.
- People with Parkinson's disease may engage in critical self-talk regarding their performance of home exercise whereby they interpret non-motor symptoms, such as lack of motivation, as personal character flaws.
- Professional guidance is essential to support and monitor digital home exercise in Parkinson's disease as this group often struggle to understand their physical limits.

## Supplemental Material

sj-pdf-1-cre-10.1177_02692155241298859 - Supplemental material for Perceptions of digitally supported home exercise for people with Parkinson's disease: A qualitative study Supplemental material, sj-pdf-1-cre-10.1177_02692155241298859 for Perceptions of digitally supported home exercise for people with Parkinson's disease: A qualitative study by Jenny Sedhed, Hanna Johansson, Elisabet Åkesson, Erika Franzén and Breiffni Leavy in Clinical Rehabilitation

sj-pdf-2-cre-10.1177_02692155241298859 - Supplemental material for Perceptions of digitally supported home exercise for people with Parkinson's disease: A qualitative study Supplemental material, sj-pdf-2-cre-10.1177_02692155241298859 for Perceptions of digitally supported home exercise for people with Parkinson's disease: A qualitative study by Jenny Sedhed, Hanna Johansson, Elisabet Åkesson, Erika Franzén and Breiffni Leavy in Clinical Rehabilitation
