# Supplementary material for: Perceptions of digitally supported home exercise for people with Parkinson's disease: A qualitative study
Source: Clin Rehabil. 2024 Dec 5;39(2):268–78. doi: 10.1177/02692155241298859 (PMC11846265; doi:10.1177/02692155241298859)
Supplement: sj-pdf-1-cre-10.1177_02692155241298859 - Supplemental material for Perceptions of digitally supported home exercise for people with Parkinson's disease: A qualitative study [file sj-pdf-1-cre-10.1177_02692155241298859.pdf]

Supplementary materials

Interview guide

Theme I: Living with Parkinson's disease

- If you were to briefly explain to someone who has no knowledge of what it means to have Parkinson's disease, what would you say?
- Do you experience any challenges in everyday life?
- Can you describe the physical symptoms that you experience?
- Do you experience any cognitive symptoms (eg. memory, fatigue, difficulty concentrating, planning, executing things, or anything else)?
- Is there anything else about Parkinson's disease that you want to highlight?

Theme II: Living a physically active life

- What are your thoughts on physical activity? What is a physically active life for you?
- Have you experienced any obstacles to live a physically active life as you would like?
- Have you thought about what motivates you to live a physically active life?
- Have your thoughts about physical activity/exercise been affected since you got diagnosed with Parkinson's disease? If so, in what way?
- What experience do you have with exercise as treatment [for disease symptoms]?
- Can you describe your previous experience with home exercise? Is there something that previously have worked well/not worked?
- Can you describe if and how your life has been affected by the pandemic? Has your access to physical activity and exercise been affected by the pandemic?
- How used are you to manage digital technology?

Theme III: The experience of exercising using technology

- Can you describe if you had any expectations for home exercise using an app?
- Did you have any concerns exercising at home with the support of an app?
- How did you experience the app and the technology? Any specific positive/negative experiences?

- Did you feel that you understood how the app and technology would be used? How easy or difficult did you find the app was to use?
- If you were to change something about the app, what would you change?
- What did you think of the follow-up calls via video link and/or telephone with the physiotherapist? Would you have liked to participate in follow-up in some other way? If so, how?

Theme IV: The experience of exercising at home using a digital tool

- Why were you interested in participating in the previous [feasibility] study?
- Did you experience anything that worked particularly well with exercising at home with the app?
- Was there something that didn't work with exercising at home with the support of the app?
- Can you tell us about how you experienced the physical [motor] exercises?
    - What did you think of them? How was they for you?
    - Was there an exercise that didn't work well for you? Can you tell me which one it was? Can you tell me why it didn’t work well?
    - Was there an exercise that you particularly liked or that worked well? Can you tell me which one it was? Can you tell me why?
    - Is there anything you would like to change to improve [the physical] exercises?
- Can you tell us about how you experienced the motor-cognitive exercises, where you performed two tasks simultaneously?
    - What did you think of them? How was they for you?
    - Was there an exercise that didn't work well? Can you tell me which one it was? Can you tell me why?
    - Was there an exercise that you particularly liked or that worked well? Can you tell me which one it was? Can you tell me why?
- What did you think of exercising 3 times a week for 10 weeks?
- Can you describe what it was like for you? How was it to schedule such exercise frequency in your daily life?
- What is your attitude towards this exercise plan?

Supplementary materials

Theme V: Experiences and insights regarding exercise at home

- Did you experience any change in your physical abilities/symptoms during or after the intervention period?
- Do you experience that your challenges in everyday life have been affected by the exercise? If so, how?
- How/What do you think about having contact with others [participants] during exercise? If you were to log in online and exercise live with others, how do you think it would have been?
- How do you experience exercise at home using the app compared to other types of exercise forms [individual/group exercise] that you have participated in?
- Was there something you lacked in the exercises that could have helped you?
- Following the intervention period, what has it been like not having access to that type of exercise? Would you consider exercising with the program again? Why/why not?
- With the aim of improving home exercise for people with Parkinson's disease, what suggestions for changes do you have? Would you agree to participate in this sort of exercise again?

Theme VI: Physical activity in daily life

- Do you experience that your physical activity has been affected by the intervention period? If so, how? Can you give examples?
- Did you make any changes in your physical activity during the intervention period, apart from the home exercise with the support of the app?
- You did set goals to be physically active during the intervention period. What was your experience setting goals?

Final questions:

- Do you think we have covered what is important to you regarding exercise for people with Parkinson's disease?
-Do you have anything else you would like to add that I haven't covered or asked about?
